# Guhong Injection Alleviates Cerebral Ischemia–Reperfusion Injury via the PKC/HIF-1α Pathway in Rats

**DOI:** 10.3389/fphar.2021.716121

**Published:** 2021-09-02

**Authors:** Li Yu, Yangyang Zhang, Xixi Zhao, Haitong Wan, Yu He, Weifeng Jin

**Affiliations:** ^1^ School of Life Sciences, Zhejiang Chinese Medical University, Hangzhou, China; ^2^ School of Basic Medical Sciences, Zhejiang Chinese Medical University, Hangzhou, China; ^3^ School of Pharmaceutical Sciences, Zhejiang Chinese Medical University, Hangzhou, China

**Keywords:** Guhong injection, cerebral ischemia-reperfusion injury, PKC–protein kinase C, HIF–hypoxia inducible factor, pathway

## Abstract

Guhong injection (GHI) is a drug for ischemic stroke created by combining safflower, a traditional Chinese medicine, and aceglutamide, a Western medicine. In this study, we investigated the curative effect of GHI on cerebral ischemia–reperfusion (I/R) injury via the PKC/HIF-1α pathway in rats. Adult male Sprague Dawley rats were randomly divided into seven groups: sham-operated, middle cerebral artery occlusion (MCAO), GHI, nimodipine injection (NMDP), MCAO + LY317615 (PKC inhibitor), GHI + LY317615, and NMDP + LY317615. After establishing an MCAO rat model, we performed neurological deficit testing, 2,3,5-triphenyltetrazolium chloride staining, hematoxylin and eosin (HE) staining, enzyme-linked immunosorbent assay, Western blotting, and q-PCR to detect the brain damage in rats. Compared with the MCAO group, the GHI and GHI + LY317615 group showed neurological damage amelioration as well as decreases in serum hypoxia-inducible factor-1α (HIF-1α), protein kinase C (PKC), and erythropoietin levels; brain HIF-1α and inducible nitric oxide synthase protein expression; and brain HIF-1α and NOX-4 mRNA expression. These effects were similar to those in the positive control groups NMDP and NMDP + LY317615. Thus, our results confirmed GHI can ameliorate cerebral I/R injury in MCAO rats possibly via the PKC/HIF-1α pathway.

## Introduction

Ischemic stroke, a serious cerebrovascular disorder, is a leading cause of death and disability globally (Wang et al., 2013; Yuan et al., 2020). Its pathogenesis includes apoptosis, inflammation, oxidative and nitrative stress, neurotoxicity, and calcium overload (Jin et al., 2017; Sun et al., 2018a; Cheon et al., 2018). Tissue plasminogen activator (tPA) is the only FDA-approved drug for acute stroke treatment; however, only <5% of patients with ischemic stroke receive tPA because it has the potential to cause hemorrhagic transformation (Chalouhi et al., 2013; Bruch et al., 2019; Kuo et al., 2020). Several recent studies have focused on finding effective medicine formulations with significant effects on ischemic stroke.

**FIGURE 1:**
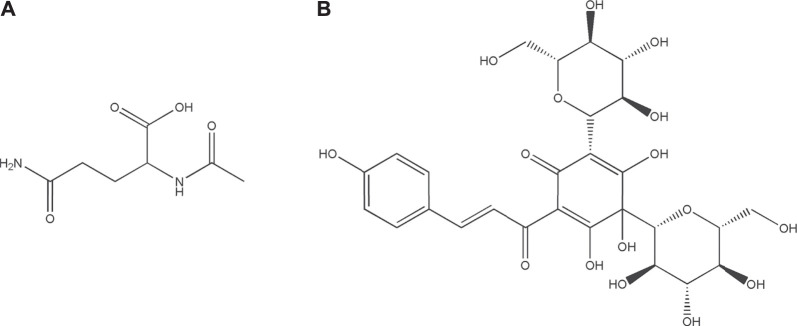
The structures of Acetylglutamine **(A)** and Hydroxysafflor yellow A **(B)**.

Guhong injection (GHI) is a typical sterile solution created by combining aqueous *Carthamus tinctorius* Linné [Asteraceae; Carthami Flos] extract (a traditional Chinese medicine) and aceglutamide (a Western medicine) (Wang et al., 2020). In other words, this is a preparation innovation for the sublimation of ethnomedicine or ethnopharmacy. We previously established GHI’s high-performance liquid chromatography fingerprint (He et al., 2015; Ai et al., 2017). GHI has been extensively applied in cerebrovascular disease treatment, mainly because of its anti-inflammatory and antioxidative effects (Ai et al., 2017; Shu et al., 2014; Zhou et al., 2021). Acetylglutamine (Figure 1A) is an acetyl derivative of glutamine—one of the most abundant free amino acids in humans (Xu et al., 2020). Aceglutamide can attenuate cerebral ischemia–reperfusion (I/R) injury (Zhang et al., 2020). *Carthamus tinctorius* Linné is commonly known as Safflower (honghua in Chinese), which is used in a dried form for treating various cardiovascular and cerebrovascular diseases clinically because its effects include blood stasis removal and meridian dredging (Zhang et al., 2016). Hydroxysafflor yellow A (Figure 1B), a main safflower component, can be used for treating ischemic diseases via platelet aggregation and thrombosis inhibition (Chen et al., 2016; Li et al., 2016). However, the mechanism underlying GHI’s effects on cerebral I/R injury has not been elucidated.

Nicotinamide adenine dinucleotide phosphate oxidase (NOX) 4 is a major reactive oxygen species source, and it plays a key role in cerebral I/R injury treatment (Pushpakumar et al., 2020). It is involved in the oxidative pentose phosphate metabolic pathway and related to myosin light-chain kinase activation (Sun et al., 2018b; Geng et al., 2019). Protein kinase C (PKC), a family of threonine serine kinases involved in many biological processes, is involved in NOX activation (Tang et al., 1994; Gulati and Singh, 2014). Hypoxia-inducible factor-1α (HIF-1α), an critical transcriptional factor regulating hypoxia and ischemia responses (Yu et al., 2020), is activated by PKC and induced hydroxylation and degradation by the enzymes prolyl hydroxylase domain (PHD) (Gibbs et al., 2011). The expression of the HIF-1α downstream gene can produce many products, including erythropoietin (EPO), NOS, EGF, PAI-1, and VEGF (Wang et al., 2019).

In this study, we investigated the pharmacological effects of GHI on cerebral I/R injury in rats and elucidated the underlying mechanism. Our results may lay an experimental foundation for clinical research on GHI in cerebrovascular disease treatment.

## Materials and Methods

### Reagents and Chemicals

GHI was procured from Tonghua Guhong Pharmaceutical (Jilin, China), nimodipine injection (NMDP) from Bayer Schering AG (Leverkusen, Germany), and 2,3,5-triphenyltetrazolium chloride (TTC) from Hangzhou Dacheng Biotechnology (Hangzhou, China). Moreover, enzyme-linked immunosorbent assay (ELISA) kits for HIF-1α, EPO, and PKC were purchased from Nanjing Jiancheng Bioengineering Institute (Nanjing, China). iNOS, PHD2, and HIF-1α antibodies were purchased from abcam Biotechnology Co., Ltd. (Hangzhou, China).

### Quality Control of GHI

According to the Chinese national standard [No. WS-10001-(HD-1506)-2004] promulgated by Chinese Pharmacopoeia Commission and the process of GHI: Each 1,000 ml of GHI contains safflower equivalent to 500 g and aceglutamide equivalent to 30 g (Ai et al., 2017). According to the corresponding quality control standard, the content of HYSA should not be lower than 0.15 mg/ml and the content of aceglutamide should be in the range of 27–33 mg/ml when detected by HPLC (Ai et al., 2017; Zhou et al., 2021). In ten batches of GHI, our research group found 21 common peaks (Ai et al., 2017). The contents of seven representative ingredients (aceglutamide, uridine, adenosine, guanosine, syringing, hydroxysafflower yellow A, and anhydrosafflor yellow B) were determined (He et al., 2015). The above studies proved that the preparation process of GHI was stable.

### Animals

Adult male Sprague Dawley rats (age = 10–12 weeks, weight = 260–300 g) were purchased from the Animal Center of Zhejiang Chinese Medicine University (laboratory animal certificate: SYXK 2018-0012). The rats were housed at room temperature with humidity 60 ± 5% under a consistent 12-h light–dark cycle with ad libitum access to food and water. All the experiment procedures were approved by the Animal Subjects Review Board of Zhejiang Chinese Medical University.

### Middle Cerebral Artery Occlusion Model Establishment

Middle cerebral artery occlusion (MCAO) method was performed according to a previously described method (Yu et al., 2018). In brief, the rats were anesthetized with 2% sodium pentobarbital (30 mg/kg) via intraperitoneal injection, and their common carotid artery (CCA), external carotid artery, and internal carotid artery (ICA) were exposed after the midline neck incision. Next, a 0.28-mm polylysine-coated nylon monofilament was inserted from the CCA into the ICA until the cerebral middle artery was reached (18 ± 2 mm). The nylon monofilament was removed after 1 h of ischemia induction. Effective establishment of MCAO was judged based on the presence of Horner syndrome in the right eye, the contralateral claw of the forelimb, and crawling in a circle to the left.

### Animal Grouping and Drug Treatment

**FIGURE 2:**
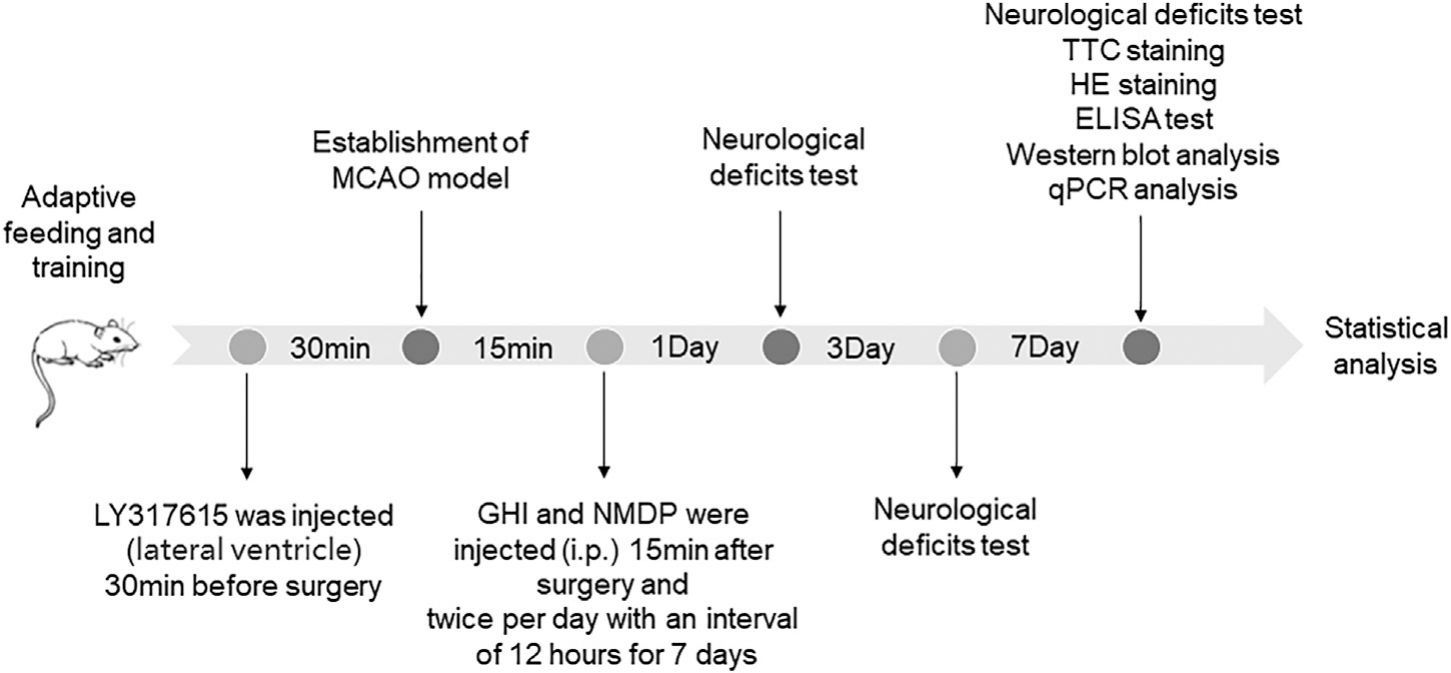
The schedule of the experimental procedures.

All rats were randomly divided into seven groups (*n* = 18 per group): sham-operated (Sham), MCAO, GHI, NMDP, MCAO + LY317615, GHI + LY317615, and NMDP + LY317615. The rats were injected with 10 ml/kg twice per day of GHI or NMDP intraperitoneally (Ai et al., 2017; Zhou et al., 2021). The dosage of LY317615 was 10 μl. The Sham and MCAO groups were intraperitoneal injected the same amount of physiological saline. All drugs or saline were administered twice per day with an interval of 12 h for 7 days. The schedule of the experimental procedures is presented in Figure 2.

### Behavioral Observations

An examiner blinded to animal treatment scored the neurological deficits 1, 3, and 7 days after MCAO by using the modified version of Garcia sensorimotor (Han et al., 2020) and Longa motor functions and neurologic (Li et al., 2020) scoring systems.

For the Garcia scoring system, spontaneous activity (0–3 points), symmetry of movements (0–3 points), symmetry of forelimbs (0–3 points), climbing wall of wire cage (1–3 points), reaction to touch on either side of trunk (1–3 points), and response to vibrissal touch (1–3 points) were evaluated. The maximum and minimum scores were 18 and 3, respectively.

In the Longa scoring system, the scores were as follows: 0 for no deficits, 1 for contralateral forepaw inflection when tail is lifted, 2 for spontaneous circling to the contralateral side, 3 for hemiplegia to the contralateral side, and 4 for inability to move spontaneously without consciousness.

### Infarct Volume Measurement

The infarct volume was determined using 2,3,5-triphenyltetrazolium chloride (TTC) staining. Rat brain samples were collected 7 days after treatment administration and immediately frozen at −20°C for 15 min. The frozen samples were then cut into 2-mm-thick coronal slices and then stained with 2% TTC solution at 37°C for 20 min in the dark. These slices were photographed, and the infarct volume was calculated using ImageJ.

### Histopathology Study

The hippocampus samples of brain tissues were collected in a cryotube with 4% paraformaldehyde after reperfusion. The samples were dehydrated and embedded in paraffin, after which coronal sections were prepared. These coronal slices were stained with hematoxylin and eosin (HE) and then used for studying histopathology. Finally, the hippocampal tissues were analyzed by acquiring images on a microscope (Eclipse TI-SR with DS-U3; Nikon).

### Serum Biochemistry

Rat blood samples were collected from the heart under deep anesthesia, and the serum samples were prepared by centrifugation at 4,500 rpm for 12 min at 4°C. The supernatant (serum) was than extracted and stored at −20°C before ELISA. Next, by using specific ELISA kits according to the manufacturer's instruction, we determined serum HIF-1α, PKC, and EPO concentrations.

### Western Blot Analysis

The protein samples of rat brain were lysed using a lysis buffer, followed by centrifugation at 12,000 rpm for 5 min at 4°C. Then, the samples were electrophoresed via the sodium dodecyl sulfate polyacrylamide gel electrophoresis and transferred onto a polyvinylidene difluoride membrane. After we added a blocking solution containing 5% skimmed milk powder with shaking for 2 h, the membrane was incubated in a primary antibody dilution buffer with shaking at 4°C overnight. This was followed by the addition of secondary antibodies and shaking for 1 h. Finally, electrochemiluminescence was observed using a chemi-capture software program.

### Quantitative Polymerase Chain Reaction

Total RNA of brain samples were extracted using TRIzol solution, followed by centrifugation at 12,000 rpm and 4°C for 10 min after chloroform addition. Next, we aspirated the supernatant. To this, we added isopropyl alcohol and centrifuged the solution at 12,000 rpm for 15 min at 4°C. The precipitate was washed with absolute ethanol to obtain RNA.

This total RNA was reverse transcribed using the HiFiScript cDNA Synthesis Kit (Kangwei Century Biotechnology). This was followed by the real-time quantitative polymerase chain reaction (q-PCR) with the following cycling parameters: 40 cycles of 95°C for 15 s and 60°C for 60 s. The relative mRNA expression levels were standardized using β-actin (the housekeeping gene). The primer sequences are listed in Table 1.

**TABLE 1:** The primer sequence.

Gene	Forward primer	Reverse primer
*Nox4*	CTC​GGC​TGA​ATC​AGA​CAG​CTA	ATT​CTT​CAG​GGC​CCC​TTT​GG
*Hif1α*	GGC​TAC​AGT​ACT​GCA​CCA​AC	GTG​CTG​TGA​TCT​GGC​ATT​CG
*β-actin*	TAC​CCT​ATT​GAG​CAC​GGC​AT	CGC​AGC​TCG​TTG​TAG​AAG​GT

### Statistical Analyses

All statistical analyses were performed on GraphPad Prism (version 8.0; GraphPad Software, San Diego, CA, United States ). Here, one-way analysis of variance was used to compare the differences between multiple groups. All data are presented as means ± standard deviations, with *p* < 0.05 considered to denote statistically significant.

## Results

### GHI Alleviated Neurological Deficits

**FIGURE 3:**
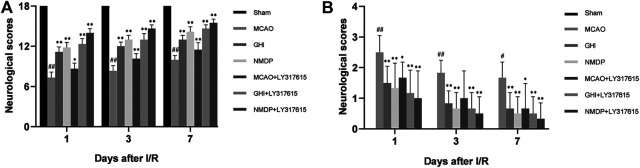
The Garcia neurological score **(A)** and Longa neurological score **(B)** in rats for each group ($\bar{x}$±SD, *n* = 6). #, compared with the Sham group; *, compared with the MCAO group; “##” and “**” means that the *p* value is less than 0.01; “#” and “*” means that the *p* value is less than 0.05.

The neurological scores were evaluated 1, 3, and 7 days after MCAO. As shown in Figure 3, the Garcia score for the Sham group was 18, indicating the absence of any neurological deficit. Moreover, compared with the MCAO group, the GHI, NMDP, MCAO + LY317615, GHI + LY317615, and NMDP + LY317615 groups showed significant improvements in neurological deficits on days 1, 3, and 7 (*p* < 0.05 or *p* < 0.01).

Based on the Longa scores (which were consistent with the Garcia scores), the neurologic deficits were the most serious in the MCAO group. Compared with the MCAO group, the GHI, NMDP, MCAO + LY317615, GHI + LY317615, and NMDP + LY317615 groups showed significant decreased in the Longa scores on days 1, 3, and 7 (*P* < 0.01), indicating that GHI could alleviate neurological deficits.

### GHI Protected Against Increased Brain Infarct Volume

**FIGURE 4:**
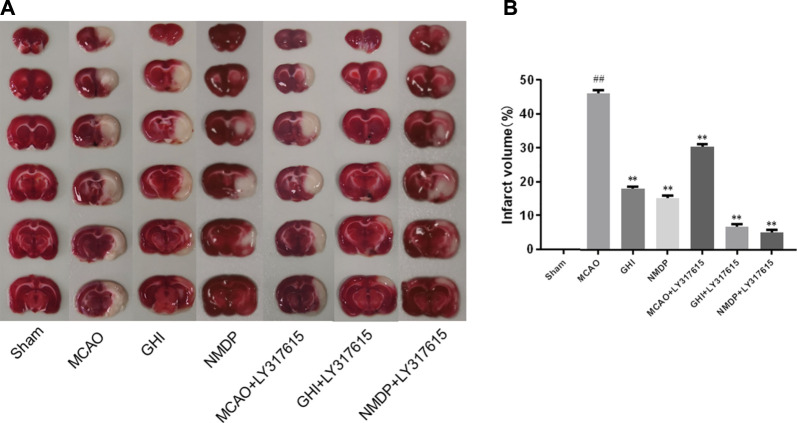
TTC staining **(A)** and infarct volume **(B)** of each group ($\bar{x}$±SD, *n* = 6). #, compared with the Sham group; *, compared with the MCAO group; “##” and “**” means that the *p* value is less than 0.01; “#” and “*” means that the *p* value is less than 0.05.

A representative photograph of TTC staining is displayed in Figure 4A, and the infarct volume in each group is presented in Figure 4B. In the Sham group, there was no infarction in rat brain. In the MCAO group, there was obvious infarction compared with the Sham group. Compared with the MCAO group, the infarct volume was significantly lower in the GHI, NMDP, MCAO + LY317615, GHI + LY317615, and NMDP + LY317615 group—indicating that GHI could alleviate the cerebral I/R injury.

### GHI Protected Neurons Against I/R Injury

**FIGURE 5:**
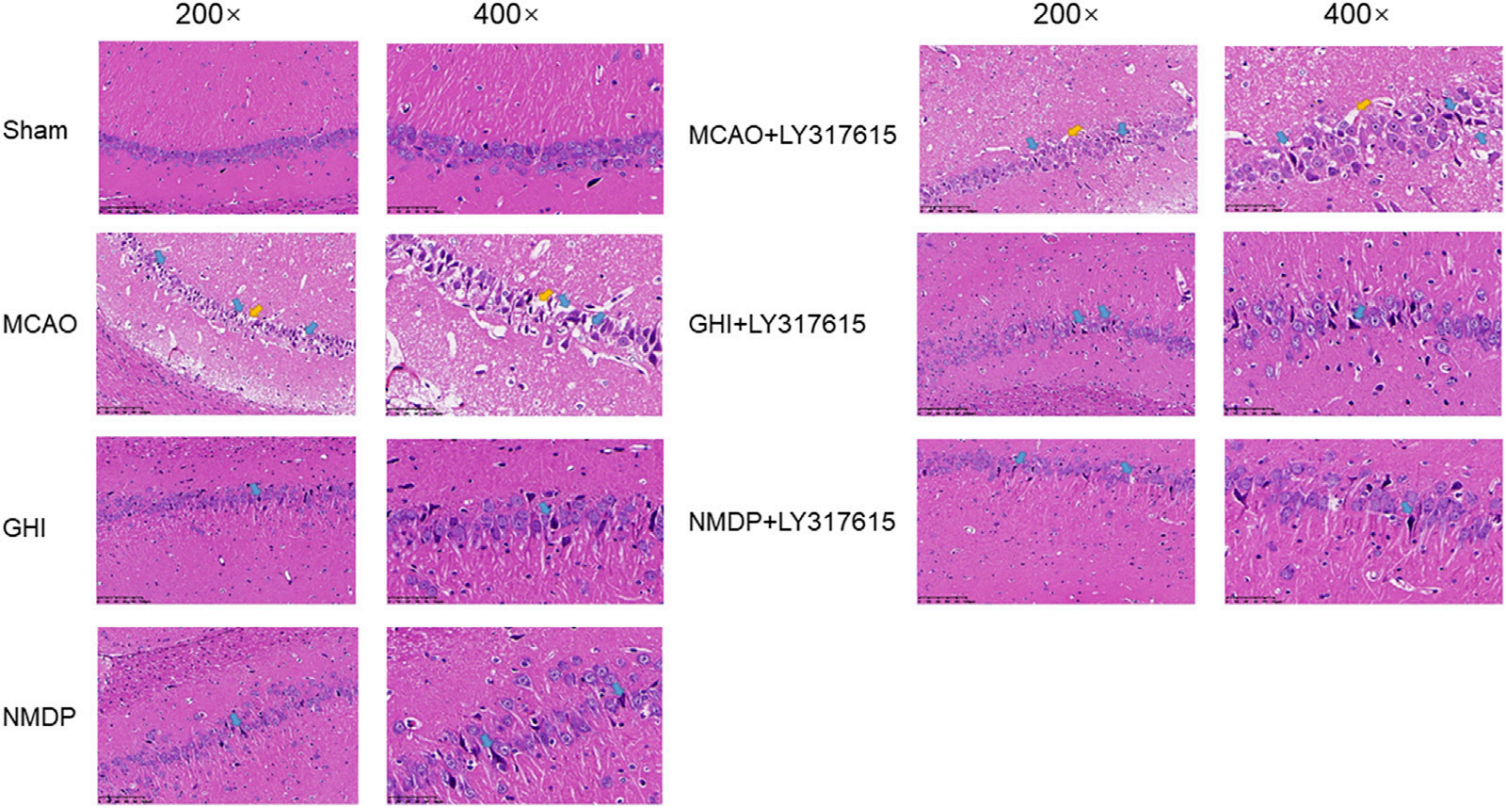
HE staining in the hippocampal for each group.

The morphological changes in the cerebral hippocampus are illustrated in Figure 5. The Sham group showed neurons arranged in an orderly manner without any necrosis. However, the MCAO group had neurons in a disordered arrangement and abnormal cell structures, such as shrunken cytoplasm and deeply stained nuclei. Moreover, the MCAO group had significantly fewer live neurons than did the Sham group. In the GHI, GHI + LY317615, MCAO + LY317615, NMDP, and NMDP + LY317615 groups, the hippocampal neurons were arranged in a more orderly manner and had more lightly stained nuclei than were those in the MCAO group.

### GHI Regulated Serum HIF-1α, PKC, and EPO Expression After I/R Injury

**FIGURE 6:**
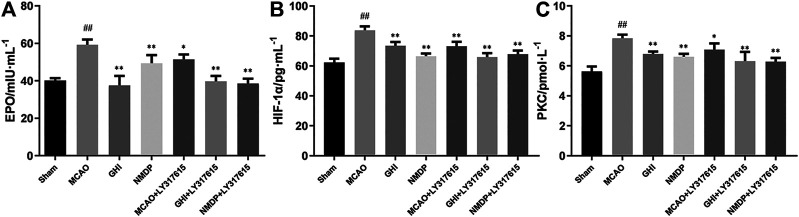
The expression of EPO **(A)**, HIF-1α **(B)** and PKC **(C)** in rat after I/R for each group ($\bar{x}$±SD, *n* = 6). #, compared with the Sham group; *, compared with the MCAO group; “##” and “**” means that the *p* value is less than 0.01; “#” and “*” means that the *p* value is less than 0.05.

Rat serum EPO, HIF-1α, and PKC expression was measured using specific ELISA kits. As shown in Figure 6, compared with the Sham group, the MCAO group significantly higher EPO, HIF-1α, and PKC levels (*p <* 0.01). In addition, compared with those in the MCAO group, EPO, HIF-1α, and PKC levels in the GHI, GHI + LY317615, MCAO + LY317615, NMDP, and NMDP + LY317615 groups were significant lower. These levels were similar to those in the Sham group.

### GHI Inhibited I/R Injury-Induced Activation via the PKC/HIF-1α Pathway

**FIGURE 7:**
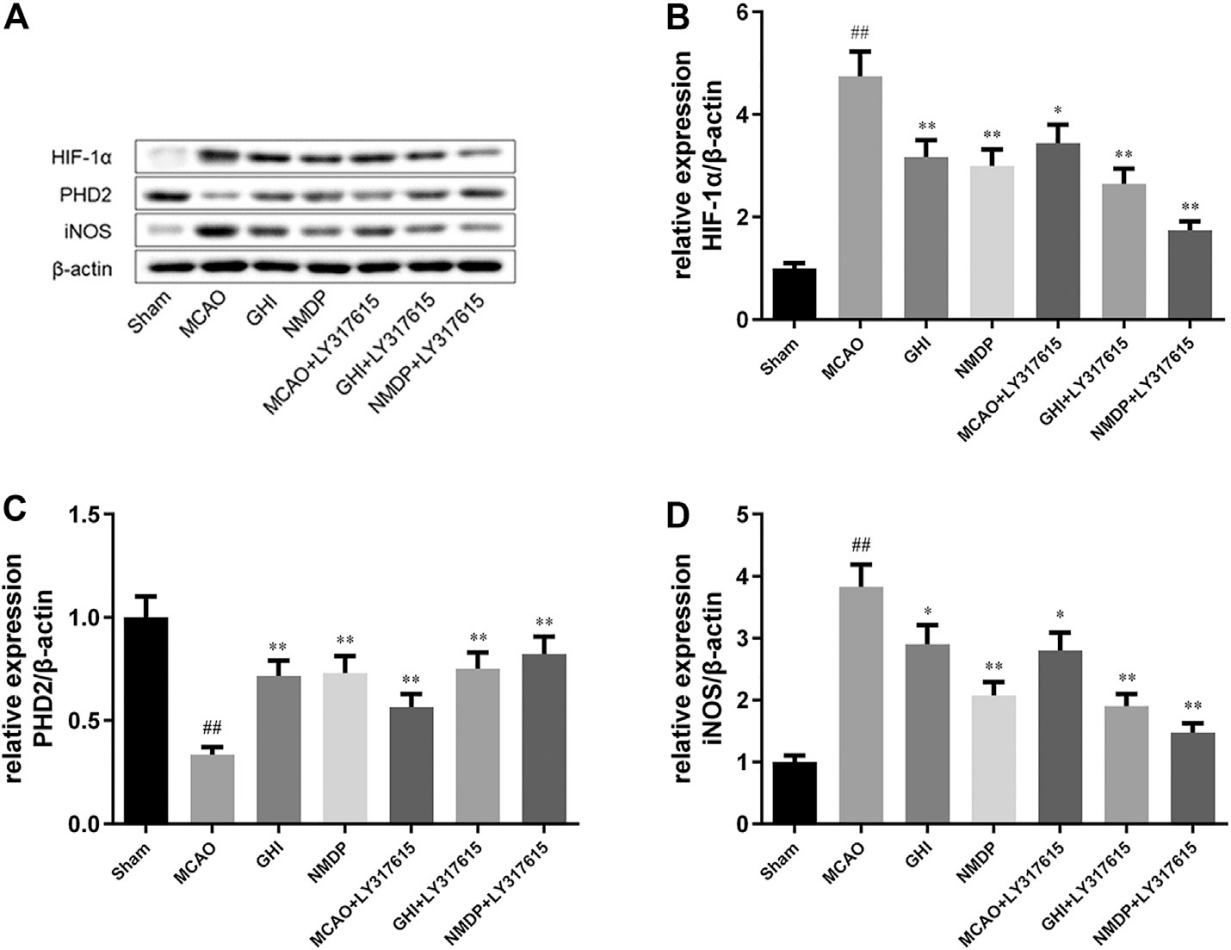
Representative photographs of Western blotting **(A)**; the expression of HIF-1α **(B)**, PHD2 **(C)**, iNOS **(D)** protein in rat after I/R for each group ($\bar{x}$±SD, *n* = 3). #, compared with the Sham group; *, compared with the MCAO group; “##” and “**” means that the *p* value is less than 0.01; “#” and “*” means that the *p* value is less than 0.05.

Representative Western blots are shown in Figure 7. Compared with the Sham group, the MCAO group showed significantly higher HIF-1α and iNOS expression and significantly lower PHD2 expression (all *p* < 0.01). Moreover, compared with the MCAO group, the GHI, NMDP, MCAO + LY317615, GHI + LY317615, and NMDP + LY317615 groups had significantly lower HIF-1 and iNOS expression and significantly higher PHD2 expression (*p* < 0.05 or *p* < 0.01). Thus, GHI alleviated cerebral I/R injury possibly via the PKC/HIF-1α pathway.

### GHI Reduced *Nox4* and *Hif1α* mRNA Expression

**FIGURE 8:**
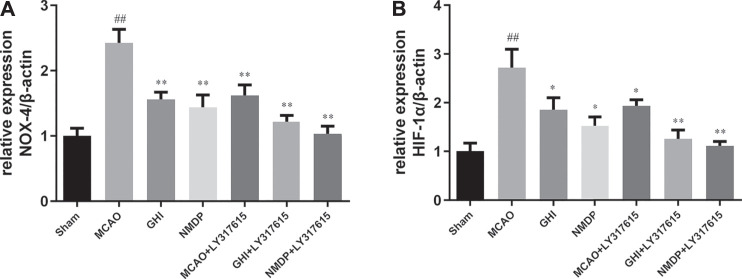
The expression of NOX-4 **(A)** and HIF-1α **(B)** mRNA in rat after I/R for each group ($\bar{x}$ ± SD, *n* = 3). #, compared with the Sham group; *, compared with the MCAO group; “##” and “**” means that the *p* value is less than 0.01; “#” and “*” means that the *p* value is less than 0.05.

As shown in Figure 8, compared with the Sham group, the MCAO group had significantly higher *Nox4* and *Hif1α* mRNA expression (*p* < 0.01). By contrast, compared with the MCAO group, the GHI, NMDP, MCAO + LY317615, GHI + LY317615, and NMDP + LY317615 groups had lower *Nox4* and *Hif1α* mRNA expression (*p* < 0.05 or *p* < 0.01).

## Discussion

Nowadays, ethnomedicine or ethnopharmacy is playing a more and more important role in the treatment of tumor, immunity, and inflammation (Michael Heinrich, 2006; Koshak et al., 2018), especially in the treatment of brain diseases.

In the recent years, ischemic stroke has caused high mortality and morbidity worldwide (Zhang et al., 2011). Cerebral ischemia leads to cognitive dysfunction and vascular dementia, which leads to increased economic and mental burden to patients and their families (Uemura et al., 2020). Studies have shown that the mechanisms underlying cerebral ischemia are complex (Liu et al., 2020; Mavroudakis et al., 2020). Moreover, traditional Chinese medicine formulations may be effect in ischemic stroke treatment (Orgah et al., 2019; Shen et al., 2020).

GHI, a formulation made by combining traditional Chinese and Western medicine components, is used for treating cerebral ischemia and coronary microvascular disease (Ai et al., 2017; Jieqin et al., 2020). We have previously studied the pharmacokinetics of the active components of GHI was investigated in rat blood and brain (Yu et al., 2018; Xu et al., 2020). Pharmacological studies have shown that GHI protects against cerebral I/R injury through anti-inflammatory and antithrombotic effects (Shu et al., 2014; Ai et al., 2017). Consistent with these studies, here we found that that GHI exerts a strong amelioration effect on cerebral I/R injury—as indicated by the reduced neurological deficit score, cerebral infarct volume, and neuronal damage. At the same time, other studies also found that GHI can protect hippocampal neurons and endothelial cells from damage by oxygen-glucose deprivation (Dai et al., 2014; Du et al., 2017; Wang et al., 2020). And these studies focus on anti-inflammatory, anti-apoptosis, anti-oxidation and other aspects of experimental research.

In the current study, the rat model of MCAO was established to explore the effects of GHI on cerebral I/R injury and understand their underlying mechanisms. First, the Garcia and Longa scoring systems were used to detected neurological deficit, with the former affording more specific results than the latter. Moreover, the Garcia score included the tests for wire cage wall climbing and tactile reflexes. Based on the results of the neurological deficit scores and TTC staining, the rats in the GHI and GHI + LY317615 groups were found to have less neurological damage and lower infarct volume—indicating that GHI had protective effects on cerebral I/R injury. HE staining showed similar results in that the GHI and GHI + LY317615 groups demonstrated significant improvements in the neuron number and morphology compared with the MCAO group. In general, GHI’s effects were similar to those of the positive control drug, NMDP.

Moreover, hypoxia is a key factor determining cell death or survival in ischemic injury. The mechanism underlying the effects of GHI on cerebral I/R injury was investigated using ELISA, Western blotting, and qPCR. The results revealed that compared with the MCAO group, the GHI and GHI + LY317615 groups had significantly lower serum HIF-1α, PKC, and EPO concentrations and brain *Hif1α* mRNA and HIF-1α expression. These results indicated that GHI could protect against cerebral I/R injury by inhibiting the PKC/HIF-1α signaling pathway. NOX4 is a major prooxidant enzyme in cerebral ischemia pathogenesis. PKC is a protein family with containing >10 isozymes, and it plays a main role in the NADPH oxidase signaling pathway, MAPK signaling pathway, and NF-κB signaling pathway (Dasu et al., 2007; Tsai et al., 2012). During prolonged periods of cerebral ischemic, PHD2 levels decrease, probably because it is the transcription target of HIF-1α. PKC inhibits PHD2 activation, whereas HIF-1α induces PHD2 hydroxylation and degradation. HIF-1α protects gene transcription by regulating adaptive responses to hypoxia and other stresses. It is crucial in the activation of endogenous substances downstream of cerebral ischemia. Studies have shown that EPO and iNOS are the critical downstream substances that are activated by HIF-1α (Wang et al., 2019). Moreover, EPO is an inducer and transducer of ischemic tolerance that prevents the death of apoptotic cells. In the current study, GHI was found to upregulate brain EPO and iNOS expression in rats with cerebral ischemia injury.

Taken together, the current results demonstrated that GHI has protective effects against cerebral ischemia via the PKC/HIF-1α signal pathway. As a formulation combining traditional Chinese medicine with Western medicine, GHI can have more multitarget pharmacological effects. Therefore, other mechanisms underlying GHI’s protective effects against cerebral ischemia warrant further investigation. Especially *in vitro* experiments, GHI needs further follow-up verification.

## Conclusion

**FIGURE 9:**
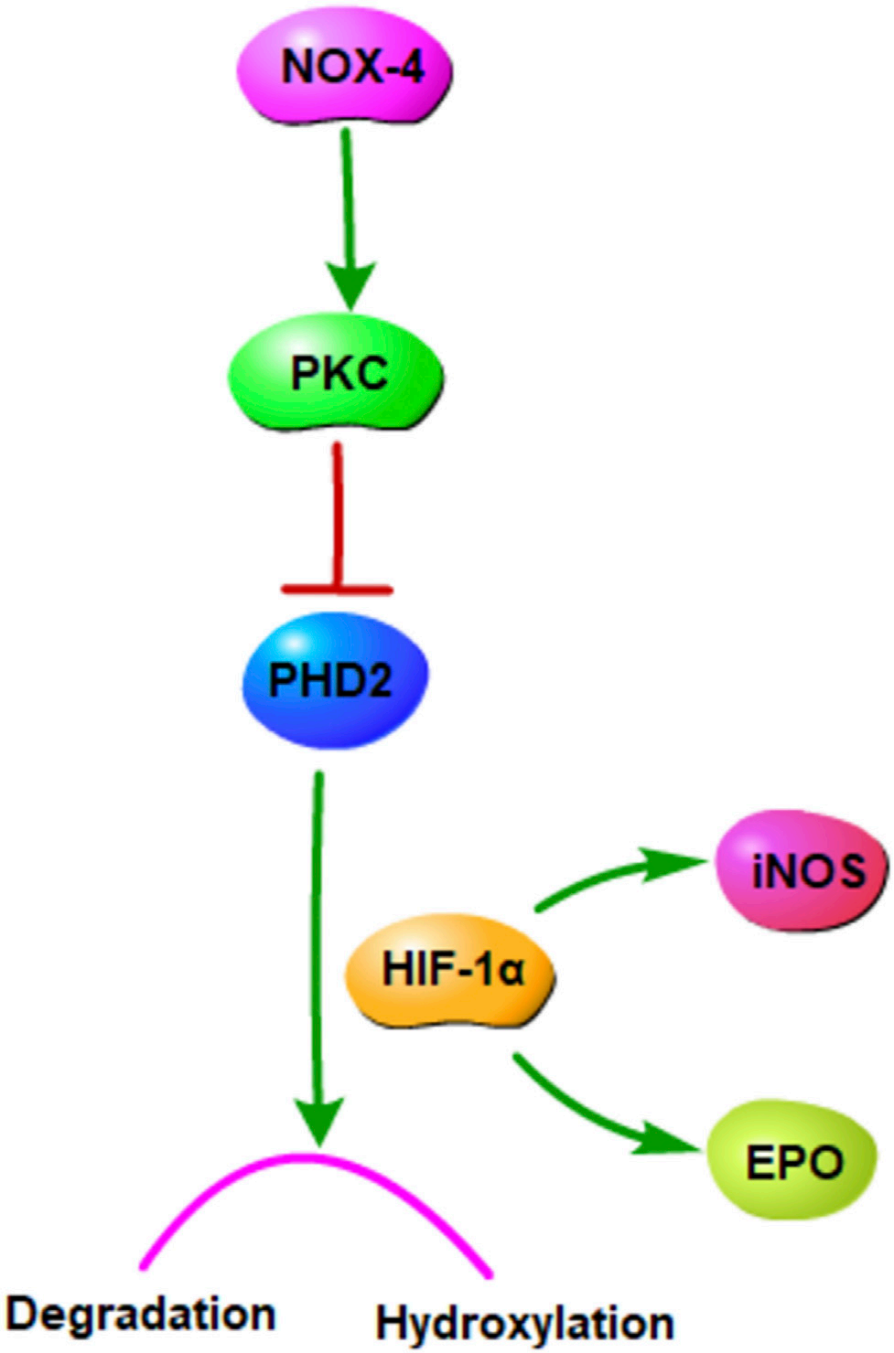
The PKC/HIF-1α signaling pathway in the condition of hypoxia.

The PKC/HIF-1α pathway in the condition of hypoxia was shown in Figure 9. Our study demonstrated that GHI alleviated cerebral I/R injury in a rodent MCAO model through reducing EPO, HIF-1α, and PKC in plasma, up-regulating PHD2 protein and down-regulating HIF-1α and iNOS protein, reducing NOX4 and HIF-1α mRNA of MCAO rats. These results indicated that PKC/HIF-1α signal pathway may be a potential mechanism which GHI alleviates cerebral I/R injury. We will further verify this mechanism through cell experiments and further study this signal pathway.

## Data Availability

The original contributions presented in the study are included in the article/Supplementary Material, further inquiries can be directed to the corresponding authors.

## Abbreviations

GHI, Guhong injection; I/R, ischemia-reperfusion; PKC, protein kinase C; HIF-1α, hypoxia-inducible factor-1α; SD, Sprague Dawley; MCAO, middle cerebral artery occlusion; EPO, erythropoietin; iNOS, inducible nitric oxide synthase; NOX-4, nicotinamide adenine dinucleotide phosphate oxidase 4; NMDP, nimodipine injection; HPLC, high performance liquid chromatography; tPA, tissue plasminogen activator; FDA, Food and Drug Administration; PHD, prolyl hydroxylase domain; EGF,epidermal growth factor; PAI-1, plasminogen activator Inhibitor-1; VEGF, vascular endothelial growth factor; TTC, 2,3,5-triphenyltetrazolium chloride; ELISA, enzyme linked immunosorbent assay; CCA, common carotid artery; ECA, the external carotid artery; ICA, internal carotid artery; HE, hematoxylin-eosin; SDS-PAGE, sodium dodecyl sulfate polyacrylamide gel electrophoresis; PVDF, polyvinylidene difluoride; q-PCR, quantitative polymerase chain reaction.
