# Supplementary figures and images for: Guhong Injection Alleviates Cerebral Ischemia–Reperfusion Injury via the PKC/HIF-1α Pathway in Rats
Source: Front Pharmacol. 2021 Sep 2;12:716121. doi: 10.3389/fphar.2021.716121 (PMC8443782; doi:10.3389/fphar.2021.716121)

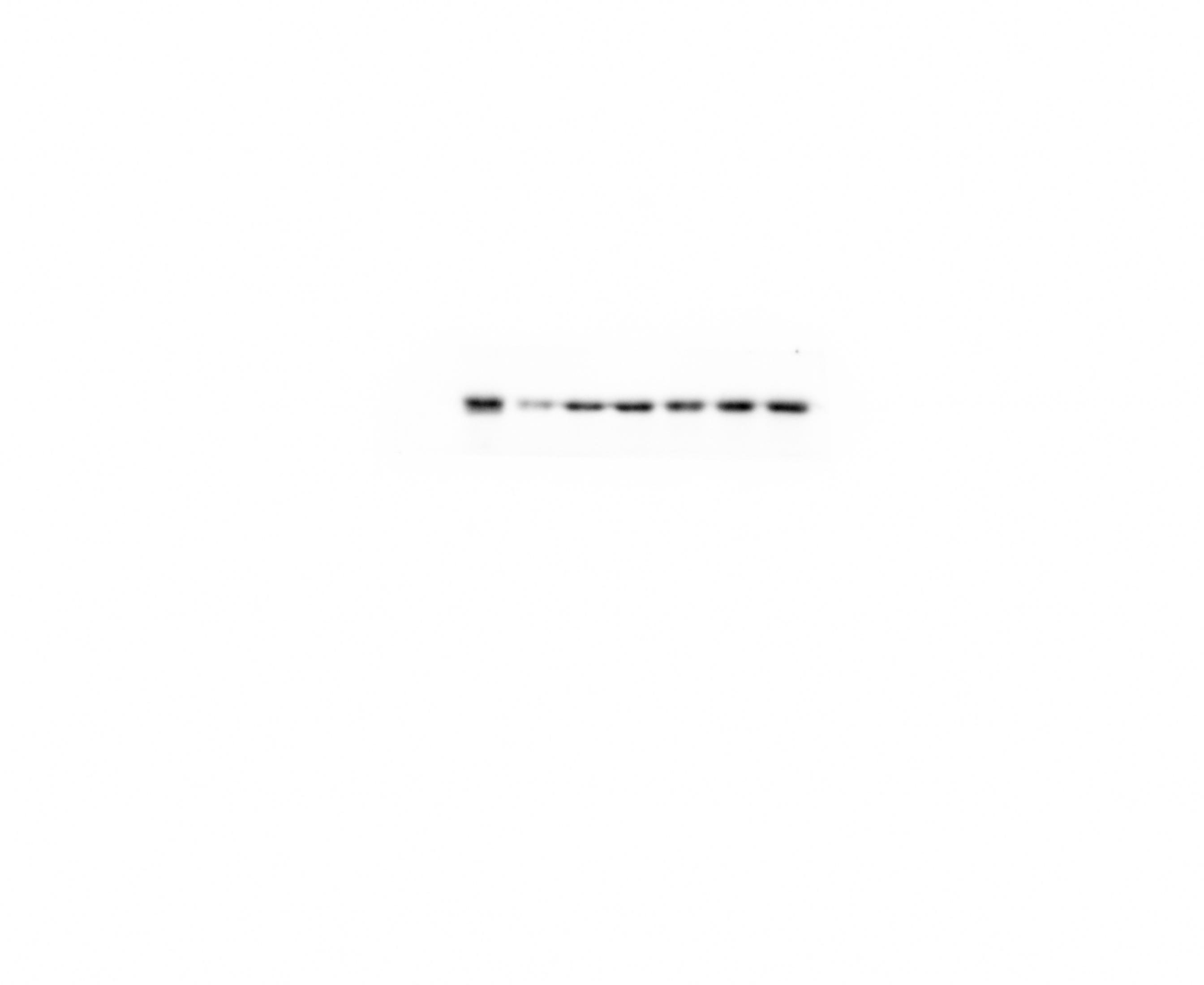

Supplement: Supplementary file 1 [file Image2.TIF]

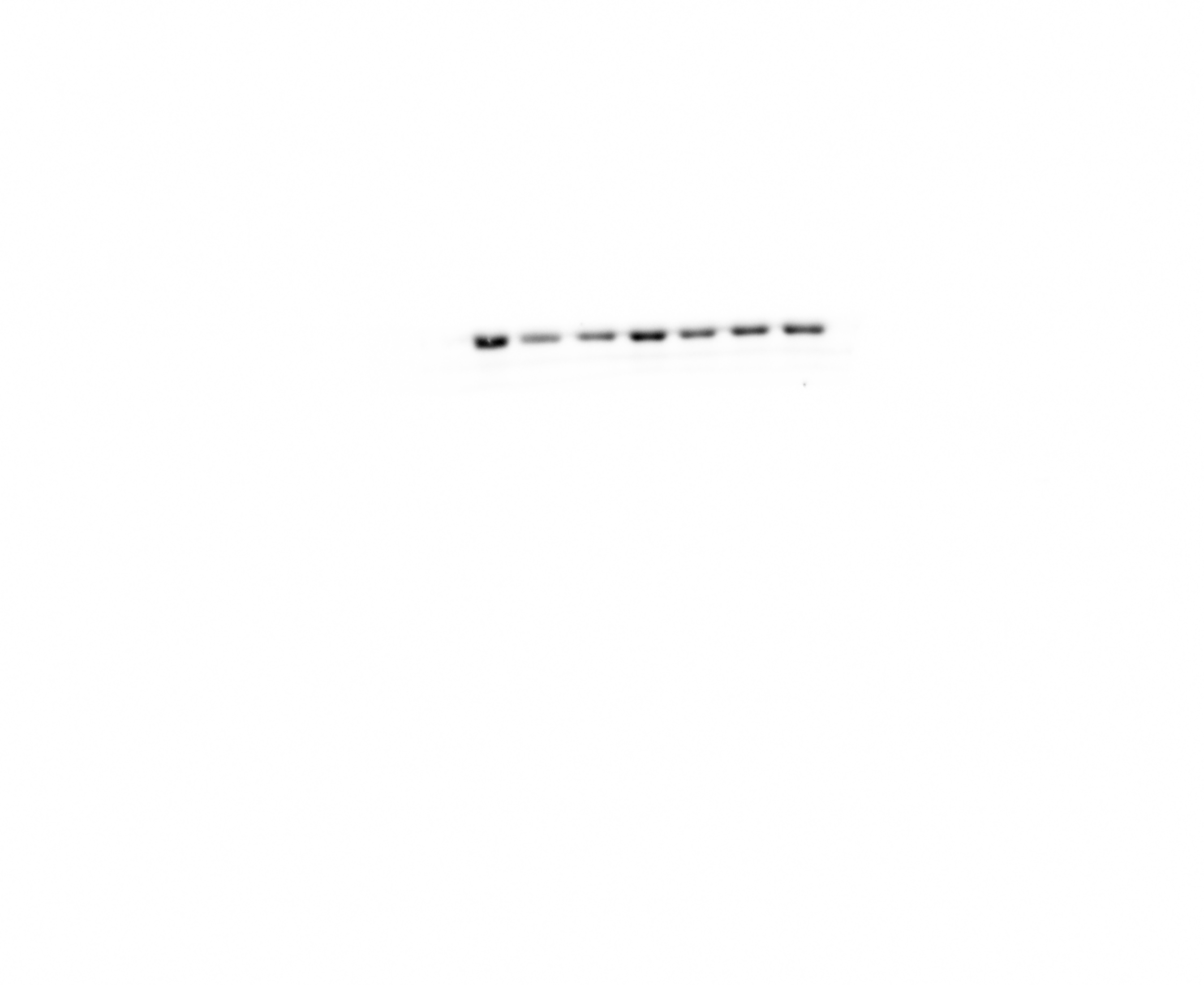

Supplement: Supplementary file 2 [file Image1.TIF]
