# Supplementary material for: Guhong Injection Alleviates Cerebral Ischemia–Reperfusion Injury via the PKC/HIF-1α Pathway in Rats
Source: Front Pharmacol. 2021 Sep 2;12:716121. doi: 10.3389/fphar.2021.716121 (PMC8443782; doi:10.3389/fphar.2021.716121)
Supplement: Supplementary file 3 [file DataSheet1.docx]

PCR:

HIF-1α：

Dissolution curve Amplification curve


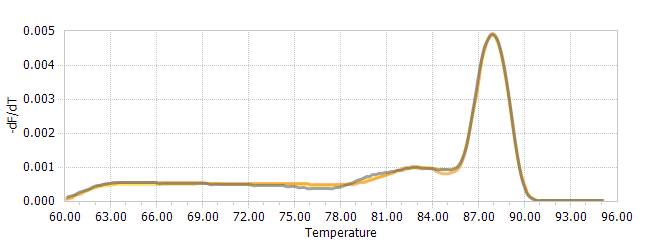

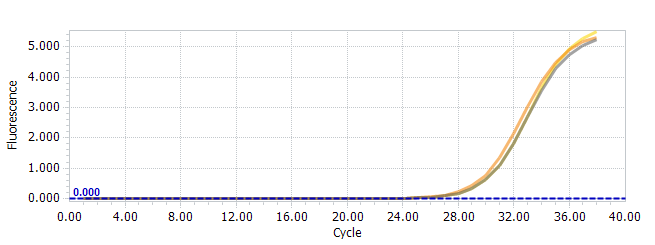


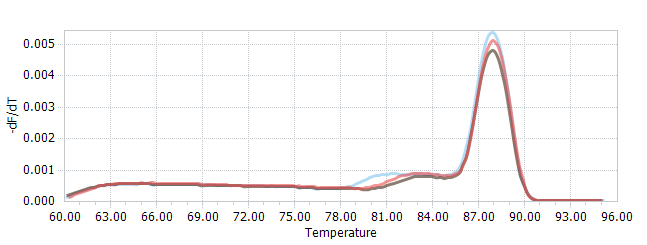

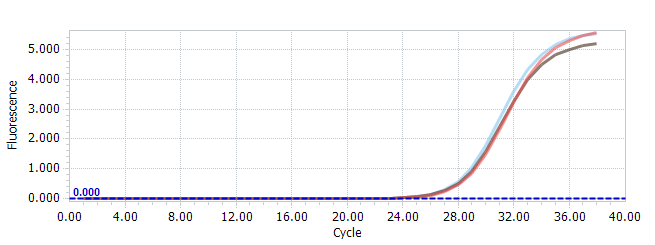


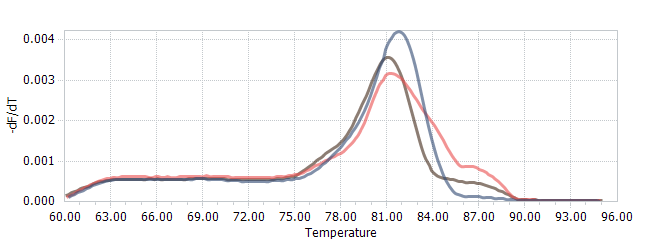

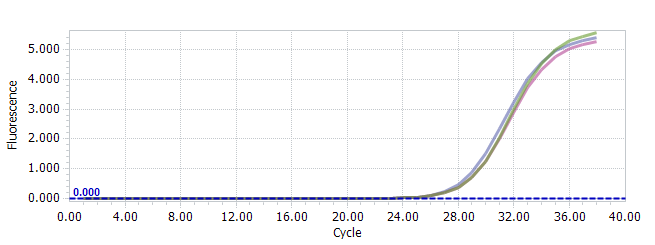


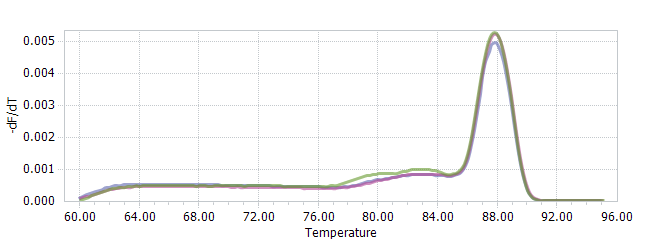

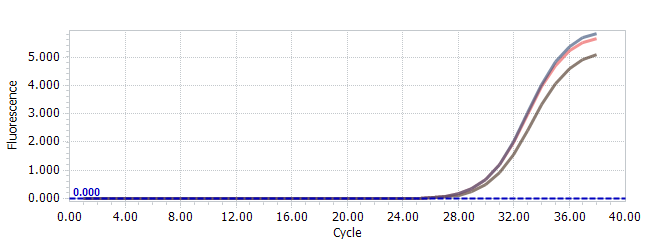


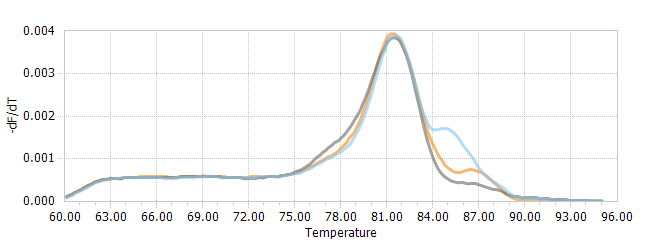

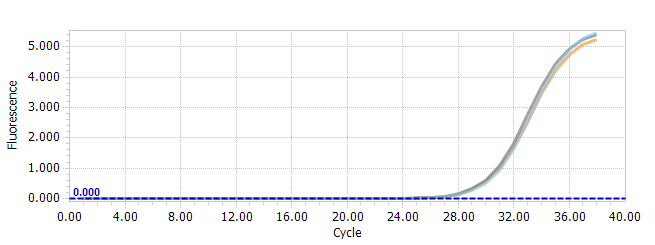


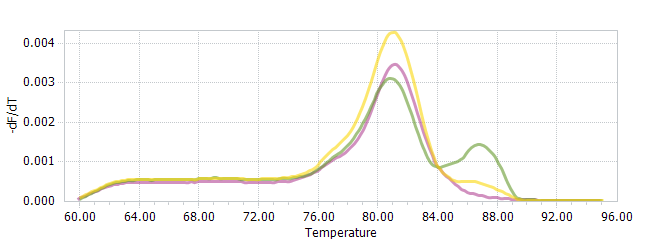

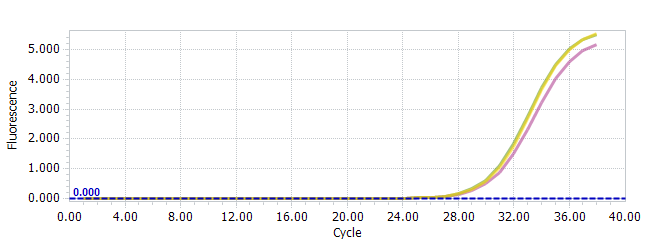


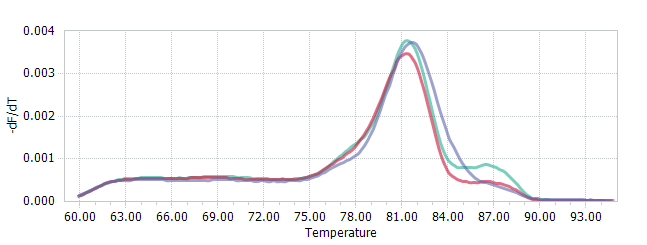

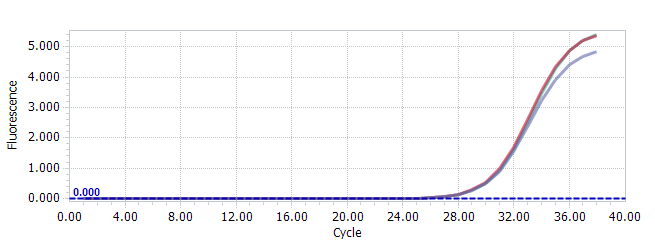


NOX-4：

Amplification curve Dissolution curve


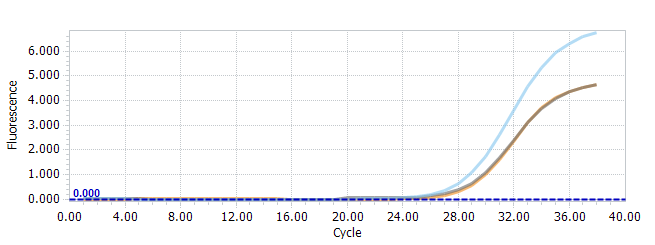

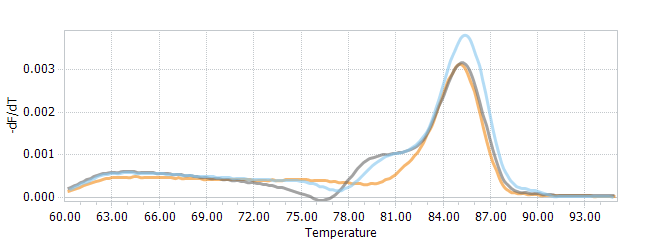


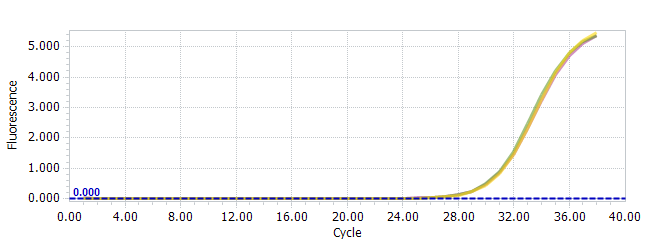

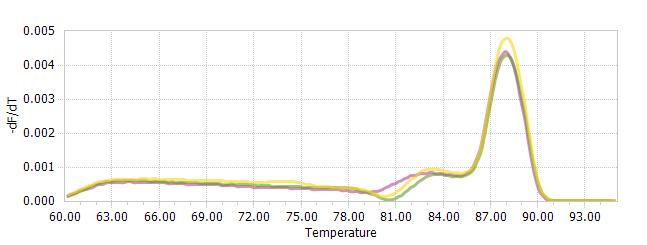


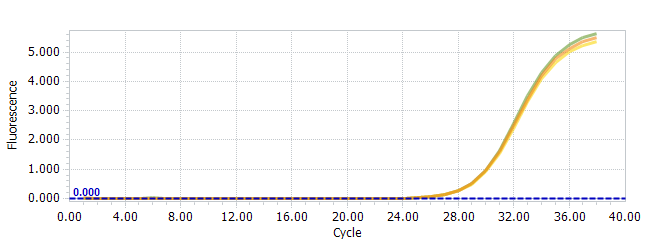

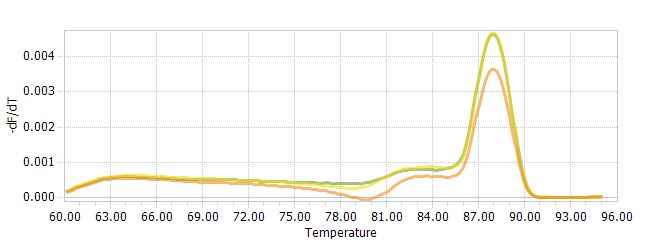


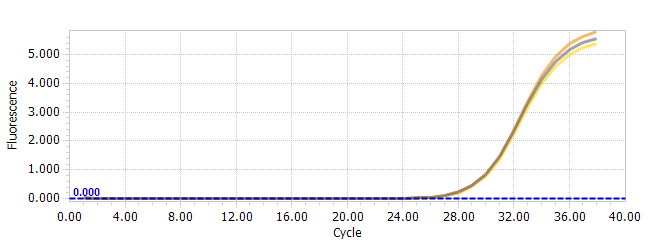

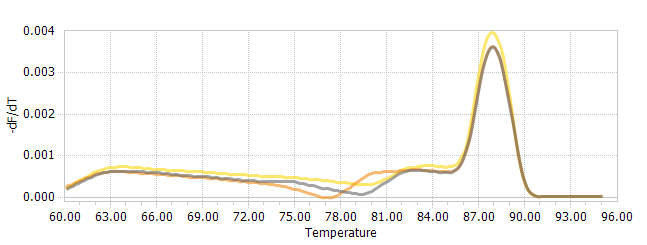


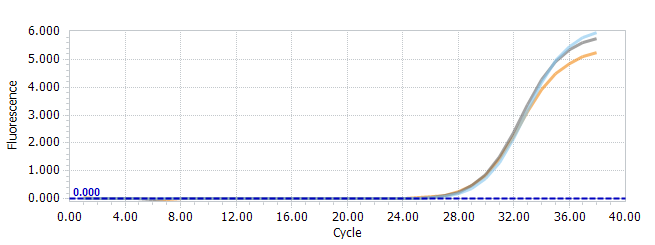

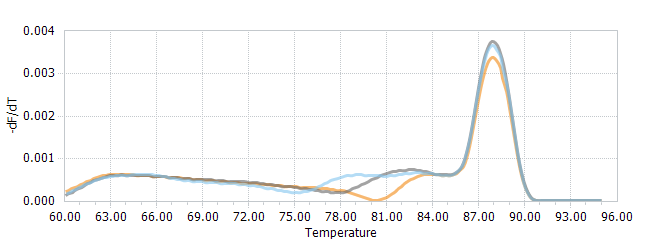


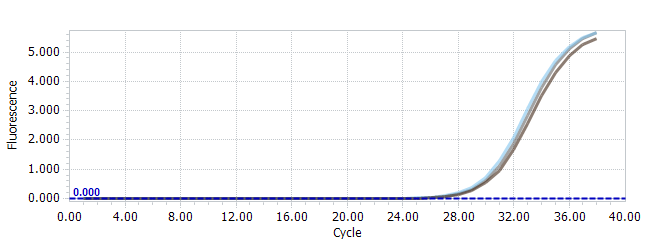

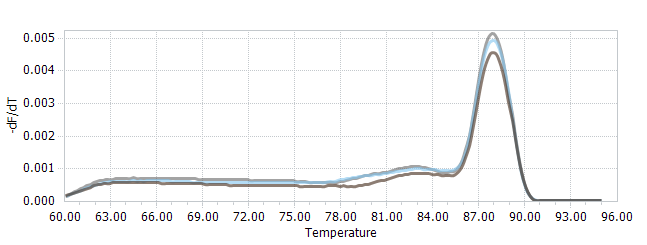


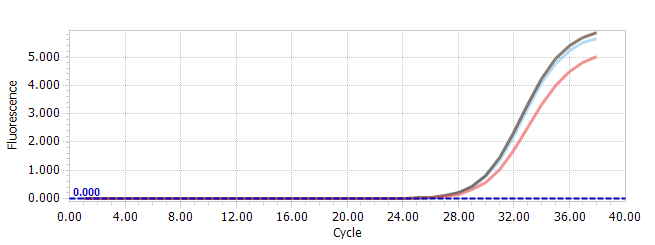

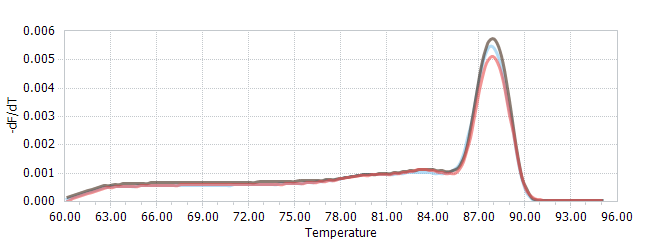


β-actin：

Amplification curve Dissolution curve


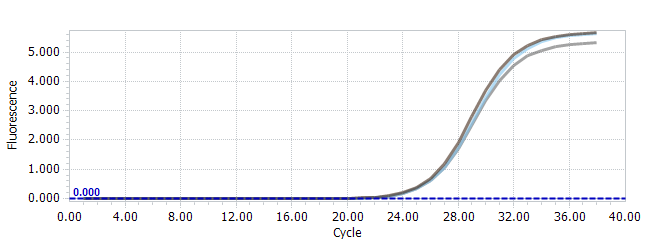

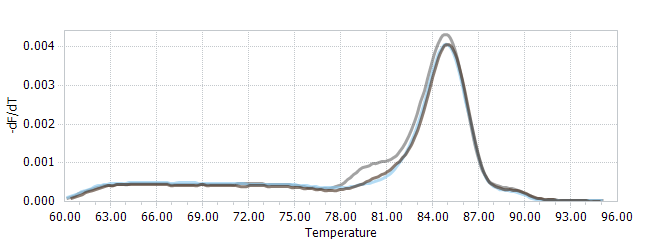


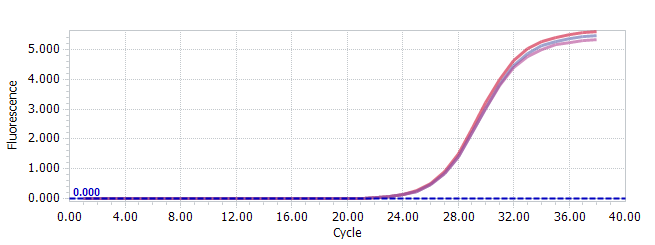

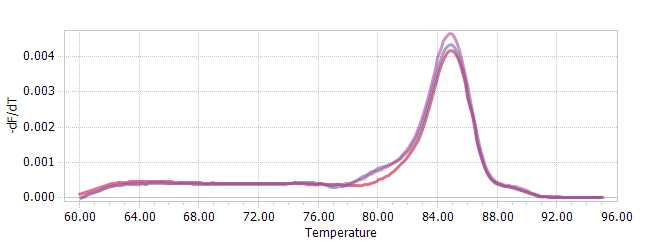


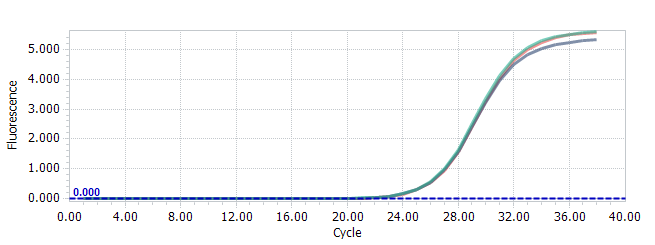

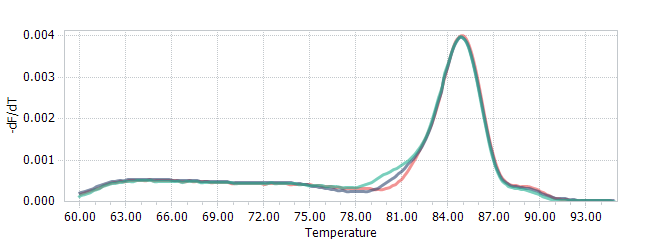


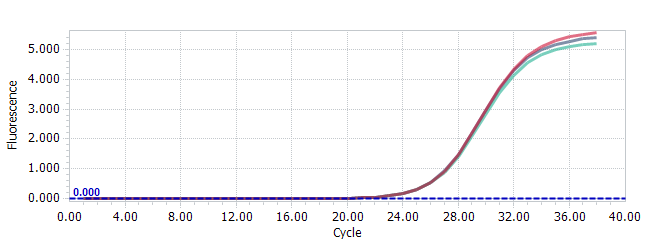

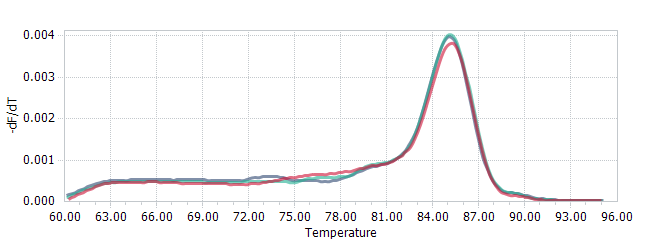


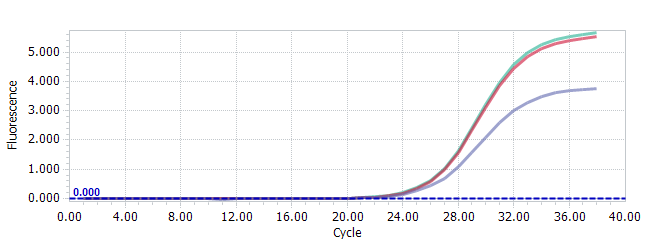

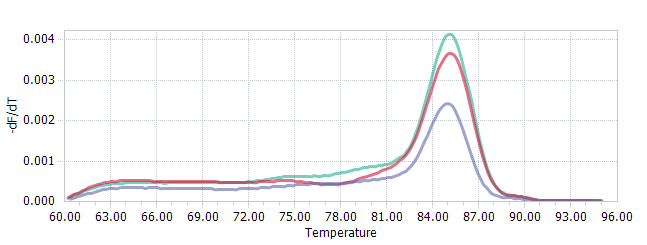


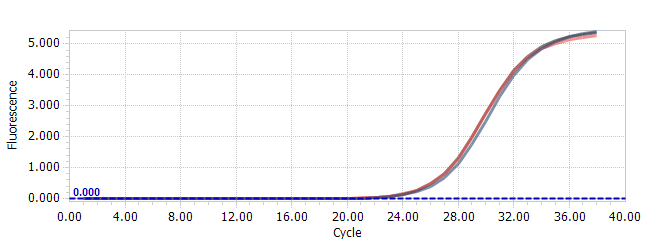

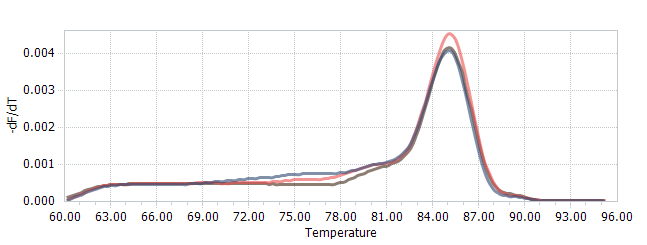


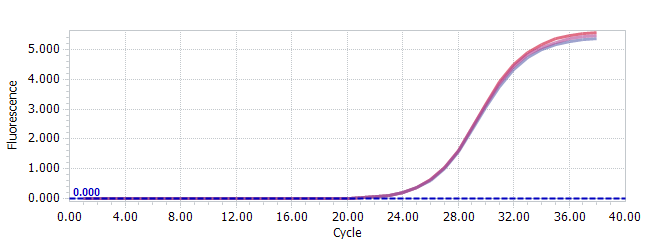

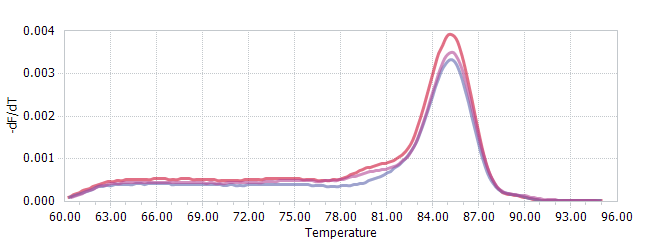


Group from top to bottom:

Sham, MCAO, GHI, NMDP, MCAO+LY317615, GHI+LY317615, NMDP+LY317615

WB：


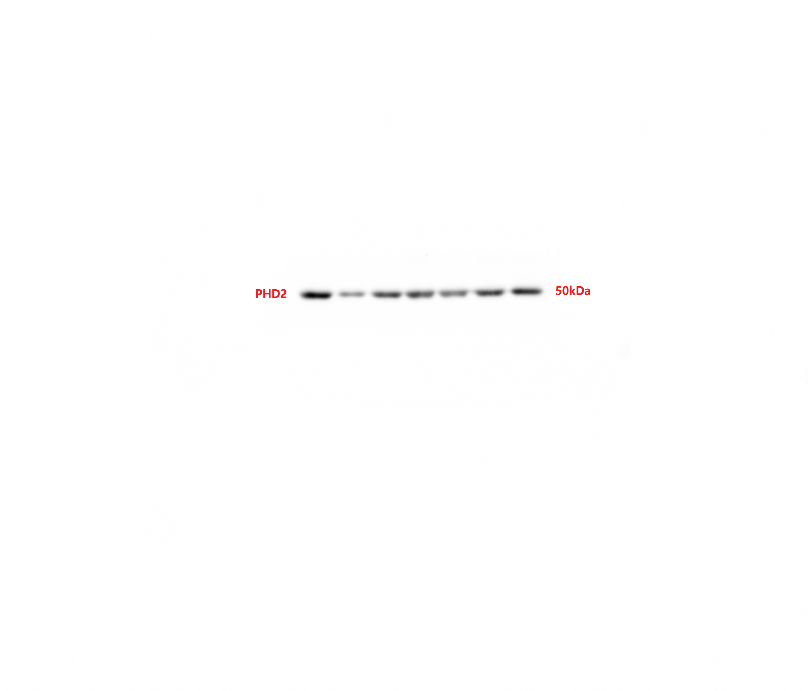

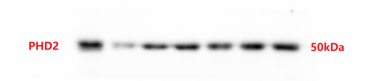

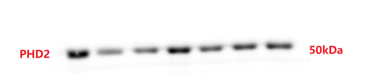


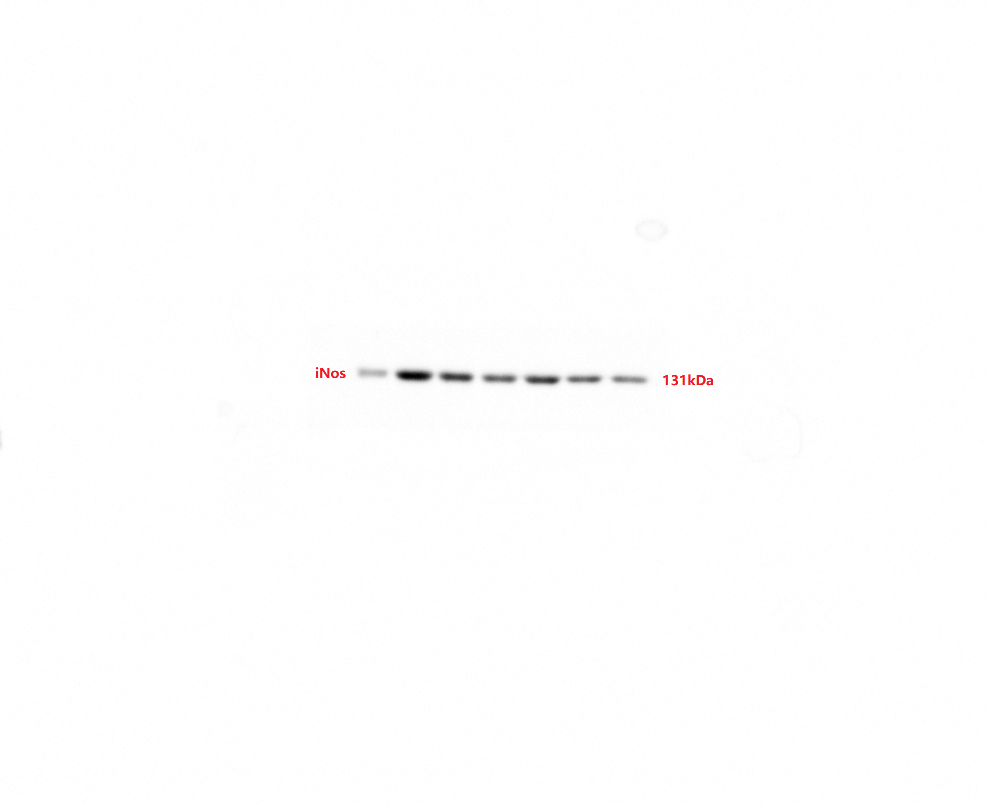

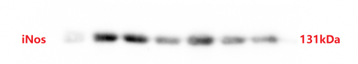

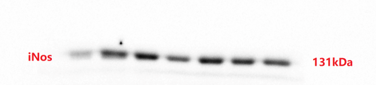


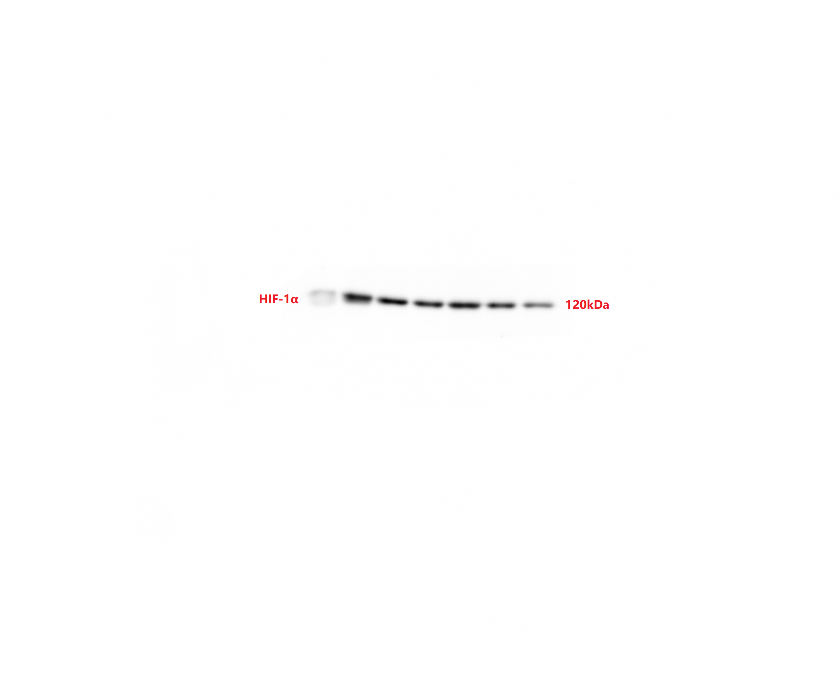

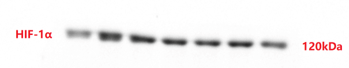

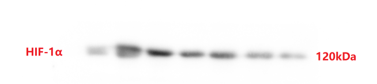


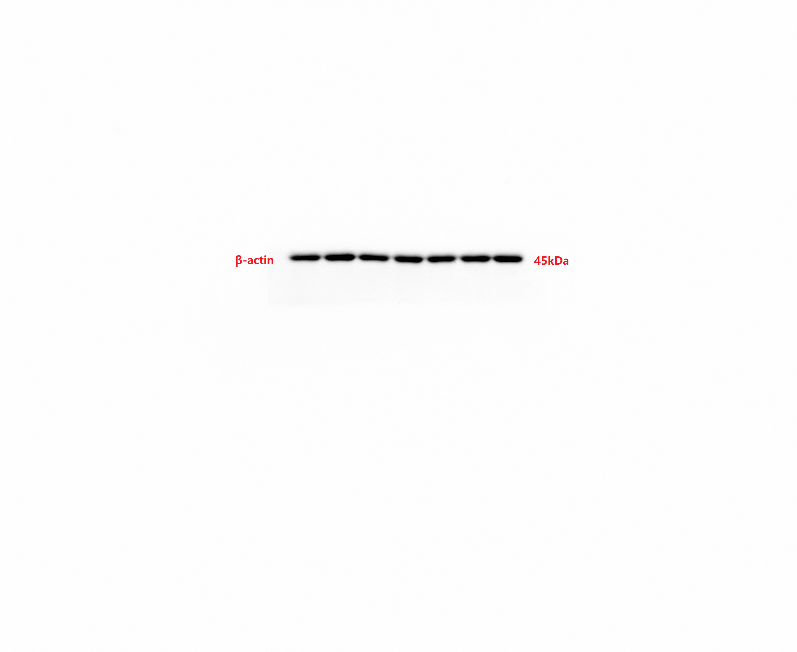

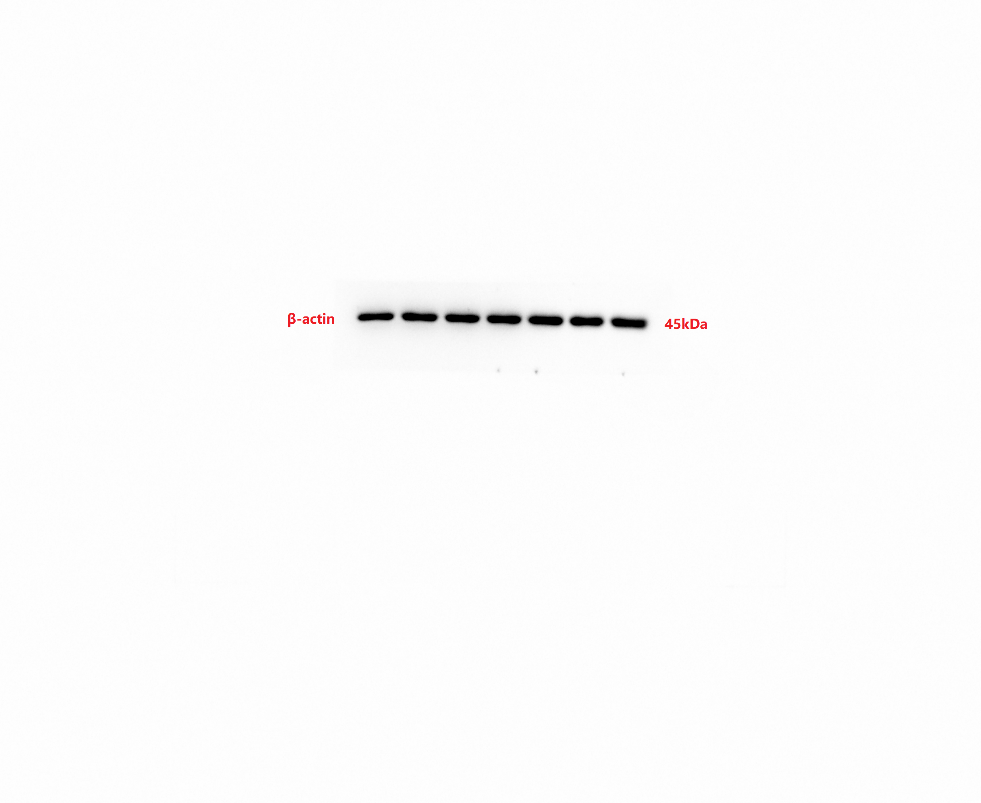

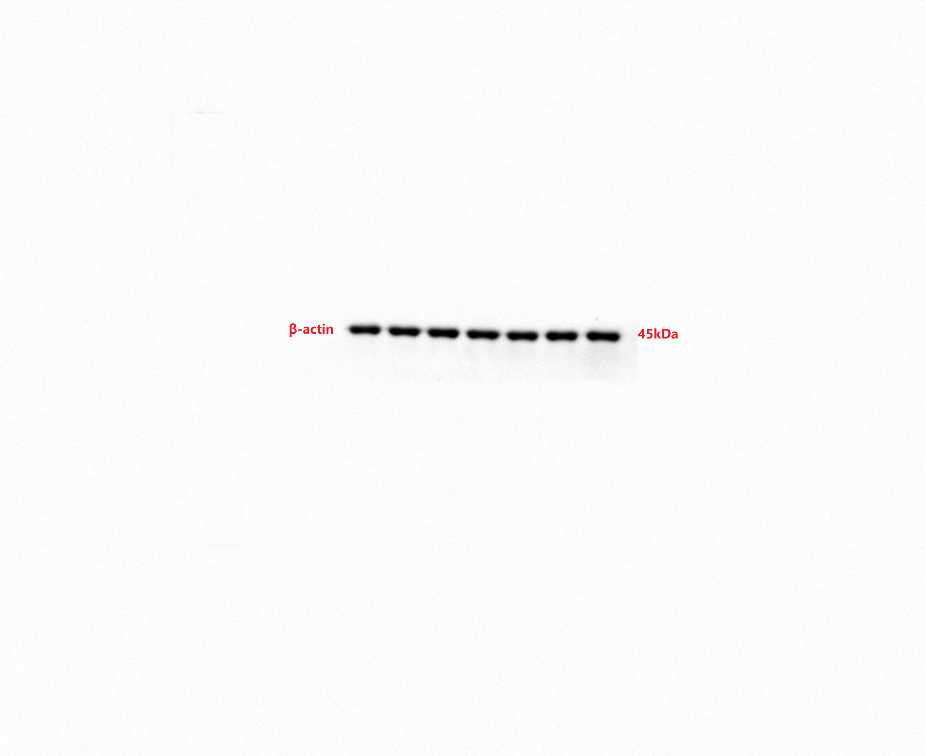


Group from left to right:

Sham, MCAO, GHI, NMDP, MCAO+LY317615, GHI+LY317615, NMDP+LY317615
